# *Streptococcus equi* subsp. *zooepidemicus*: Epidemiological and Genomic Findings of an Emerging Pathogen in Central Italy

**DOI:** 10.3390/ani15101351

**Published:** 2025-05-08

**Authors:** Francesca Cito, Cristina Esmeralda Di Francesco, Daniela Averaimo, Alexandra Chiaverini, Alessandra Alessiani, Marco Di Domenico, Marta Cresci, Marco Rulli, Maria Chiara Cantelmi, Maria Daniela Di Bernardo, Angelo Giammarino, Giacomo Vincifori, Antonio Petrini

**Affiliations:** 1 Istituto Zooprofilattico Sperimentale dell’Abruzzo e Molise—IZSAM, 64100 Teramo, Italym.cresci@izs.it (M.C.); a.petrini@izs.it (A.P.); 2 Faculty of Veterinary Medicine, University of Teramo, 64100 Teramo, Italy; cedifrancesco@unite.it; 3 Freelance Veterinary Practitioner, 66023 Chieti, Italy; 4 Unità Operativa Complessa Servizio di Sanità Animale, Azienda USL di Pescara, 65124, Pescara, Italy

**Keywords:** *Streptococcus equi* subsp. *zooepidemicus*, One Health, whole-genome sequencing

## Abstract

*Streptococcus equi* subsp. *zooepidemicus* (SEZ) is a major problem in equine veterinary medicine. Typically, a commensal in horses, SEZ can cause severe disease including respiratory infections, septicaemia and reproductive tract infections under certain conditions. Recent evidence suggests that humans can also develop severe disease infection through direct contact with infected animals or the consumption of contaminated unpasteurised milk and milk products. This study investigates SEZ strains isolated from nasal swabs of equidae in central Italy in 2023 to describe the epidemiology and genomic characteristics of circulating strains. A sampling plan was implemented to randomly collect nasal swabs from equid farms in the Abruzzo and Molise regions. In addition, a sampling form was designed to collect information on risk factors related to the presence of the bacterium and the potential development of respiratory disease. Relative risk was used to measure the association between the presence of SEZ in the samples and various variables. The swabs were analysed by real-time PCR and isolation. To confirm the identification and characterise the strains, the isolates were fully sequenced by next-generation sequencing (NGS) using the Illumina platform. A total of 478 animals from 99 farms were sampled and 30% of the animals tested positive for SEZ (79% horses, 20% donkeys, 1% mules). Forty-five percent of the farms tested positive for SEZ. Monitoring the clonal spread of SEZ is essential to understand the ecology of this emerging zoonotic pathogen, to assess the risk, and to implement effective control measures. In addition, genomic assessments are recommended to investigate the pathogenicity of circulating strains. This study provides a comprehensive understanding of the epidemiology and genomic diversity of SEZ strains isolated in central Italy.

## Simple Summary

*Streptococcus equi* subsp. *zooepidemicus* (SEZ) is a commensal bacterium of equids that can sometimes cause severe respiratory, reproductive, and even septicaemic disease; recently, it has been suggested that SEZ may even be responsible for human infections. The aim of this study was to describe the epidemiology and genomic diversity of SEZ strains by the isolating and sequencing of bacteria from nasal swabs of equids from central Italy (Abruzzo and Molise). Potential risk factors associated with the development of respiratory disease in equids were also considered.

## 1. Introduction

*Streptococcus equi* subsp. *zooepidemicus* (SEZ) is a commensal bacterium found on the skin and mucous membranes of horses. As an opportunistic pathogen, it can cause a variety of infections, including those of the respiratory tract and the reproductive and urinary tracts. These infections result in significant morbidity, economic loss, and complications in equine health management. Although SEZ can cause clinical respiratory disease in horses, not all exposed horses become ill. Horses carrying these bacteria can still infect other horses, even if they do not show any clinical signs of disease.

Recent evidence suggests that SEZ may also cause severe diseases in humans following contamination by infected animals or the consumption of unpasteurised milk and milk products [1,2,3]. Human infection with SEZ can result in symptoms ranging from mild skin infections to meningitis and sepsis, particularly in people with weakened immune systems [4,5,6]. Respiratory symptoms, including sore throat and pneumonia, can occur, especially if the bacteria are spread by direct contact with infected animals [7]. A large SEZ outbreak with 37 clinical cases was reported in Italy between November 2021 and May 2022 [8].

SEZ has also recently been identified as the causative agent of severe cases of pneumonia in donkeys in Italy, where a novel SEZ sequence type (ST525) was responsible for the death of four donkeys raised on a farm between March and April 2022 [9].

As a commensal bacterium, there is limited information available on its actual distribution. In a study by the United States Department of Agriculture, bacterial cultures from nasal swabs of 6000 healthy horses showed a SEZ prevalence of 9.2% [10].

In addition, little is known about the genetic factors that distinguish non-pathogenic strains from pathogenic strains, in both horses and humans.

The aim of this study was to assess the prevalence of SEZ at both individual and herd level in the Italian regions of Abruzzo and Molise using a cross-sectional study of nasal swab samples. The study also aimed to identify risk factors associated with the disease in horses and to characterise SEZ using whole-genome sequencing.

## 2. Materials and Methods

### 2.1. Study Area and Target Population

Regulation (EU) 2016/429 [11] requires the Member States of the European Union to establish a computerised database for terrestrial animals, including equidae. Following the entry into force of the above-mentioned law and related regulations, Italy implemented them with Legislative Decree No. 302 of 30 September 2021 and Legislative Decree No. 134 of 5 August 2022 [12,13], which establish the identification and registration system and define the management and operation of the national equine registry. The Italian Equine Register allows the search of individual equine information by unique identifiers such as the Single Lifetime Identification Document (SLID—also known as the passport), the Unique Equine Life Number (UELN) code, the transponder code, or the name of the animal. Through the specific interface with personal records, it is possible to visualise the above codes together with details of the species, breed, sex, date of birth, and whether or not the individual animal is intended for food production.

According to official data from the Italian equine register, 474,759 equines were registered in the database on 31 December 2023, of which 18,933 (4%) were in Abruzzo and 4657 (1%) in Molise. The median number of animals per farm in the study population was 44. Of the farms in the areas included in the analysis, 43% had only 1 animal, 48% had up to 10 animals, 5% had between 11 and 20 animals, and 5% had more than 20 animals.

### 2.2. Sampling Design and Collection

A sampling plan was designed to collect nasal swabs from equidae randomly distributed in the regions of Abruzzo and Molise. To define the sample, the animals were considered as the sampling units, with an assumed a priori prevalence of 50%, a confidence level of 95%, and an absolute accuracy of 5%. Based on these parameters, the minimum required number of primary units required was 384 animals. Approximately 25% additional animals were sampled to provide a safety margin, bringing the total number of primary units to 478. Sampling parameters were defined based on the number of holdings with at least one animal (*n* = 5050) registered in the National Equine Register.

A model accompanying form was designed to collect anamnesis information on the sampled animals, such as the animal’s unique identifier, species (equine, asinine, or mule), age, sex, and breed, if intended for food production, and the presence of respiratory symptoms. In addition, information on the farm was collected such as the unique identification number, the species farmed, the type of activity carried out on the farm with animals of the same species or group of species, geographical location, and the name of the farm operator. Where information was missing from the sample accompanying form, it was obtained by consulting the National Equine Register.

Veterinarians collected samples from the nasal cavities of 478 adult horses using sterile swabs. The nostrils were cleaned with paper towels before the distal nasal cavity was swabbed, and then the swabs were placed in a transport medium for delivery to the laboratory. The. Istituto Zooprofilattico Sperimentale (IZS) in Teramo, Italy, performed the tests.

### 2.3. Data Management and Statistical Analysis

The unique identifier of the animal, the species sampled and the unique registration number of the facility reported on the form were also registered in the Laboratory Information System (LIMS) of the IZS Teramo. At the end of the registration process, a label with a sequential number was generated to uniquely identify the nasal swab collected from each animal. A database was created using data extracted from the LIMS, together with additional information collected from accompanying forms or obtained from the National Equine Register.

Each entry of animal, farm and laboratory into the database was double checked to ensure data quality. The spatial location of each sampled establishment, intended as the physical location where animals are kept, was plotted on a map using Geographic Information System (GIS) software, (QGIS 3.34.12-Prizren.QGIS.org, 2024. QGIS Geographic Information System. QGIS Association. http://www.qgis.org, accessed on 23 April 2025).

The prevalence of SEZ was calculated at both animal and farm level, with a 95% confidence interval determined using exact binomial confidence intervals.

Multivariate statistical analysis was performed using multivariate logistic regression, with the response variable being the presence or absence of SEZ on the collected swab, and the covariates including sex, species, breed, age category (less than or greater than 15 years, with 15 years serving as the threshold for classifying a horse as “old” [14]), symptoms, whether the animal was intended for food production, the simultaneous presence of multiple livestock species, and the presence of dairy species on the same farm. The potential relationship between the presence of SEZ and the covariates included in the model was also assessed using a chi-square test. A two-tailed Mann–Whitney test was used to assess age differences between negative and positive animals. All statistical analyses were performed using R v.4.3.2 (R Development Core Team, 2023).

### 2.4. Isolation, PCR, and Sequencing of SEZ

For isolation, nasal swabs were plated on 5% sheep blood agar plates (Microbiol srl, Cagliari, Italy) and incubated for 24–72 h at 37 ± 1 °C in a 5–10% CO_2_-enriched atmosphere. Suspect *Streptococcus* spp. colonies were subcultured and identified by MALDI-TOF (MALDI Biotyper^®^, Bruker Daltonics Gmbh & Co. KG, Bremen, Germany). DNA was then extracted from isolated colonies for sequencing using the Maxwell^®^ RSC Genomic DNA Kit (Promega, Fitchburg, WI, USA) with minor protocol modifications. Nasal swabs were also subjected to DNA extraction using the Maxwell^®^ RSC Genomic DNA Kit, followed by real-time PCR testing for SEZ using a commercial kit (Genesig^®^ Primerdesign™ Ltd., Manchester, UK), targeting the SDR family oxidoreductase gene. The kit was validated prior to use and demonstrated 100% sensitivity and specificity (95% CI: 86.7–100%).

DNA extraction was performed with minor modifications for a total of 51 strains and NGS was performed using the Illumina platform (Illumina, San Diego, CA, USA). An in-house pipeline (https://github.com/genpat-it/ngsmanager/, accessed on 10 September 2024) was used for WGS data analysis and a quality check was performed. The KmerFinder tool [15] was used to confirm species identification.

Multilocus Sequence Typing (MLST) was performed according to the reference scheme (https://pubmlst.org/organisms/streptococcus-zooepidemicus, accessed on 15 September 2024). Virulence profiles were obtained using ABRicate (https://github.com/tseemann/abricate, accessed on 15 September 2024).

To verify the correlation between the 51 SEZ genomes, a single-nucleotide polymorphism (SNP) analysis was performed using the CFSAN pipeline [16] with CP001129 (*Streptococcus equi* subsp. *zooepidemicus* MGCS10565) as a reference.

## 3. Results

### 3.1. SEZ Prevalence

**Figure 1:**
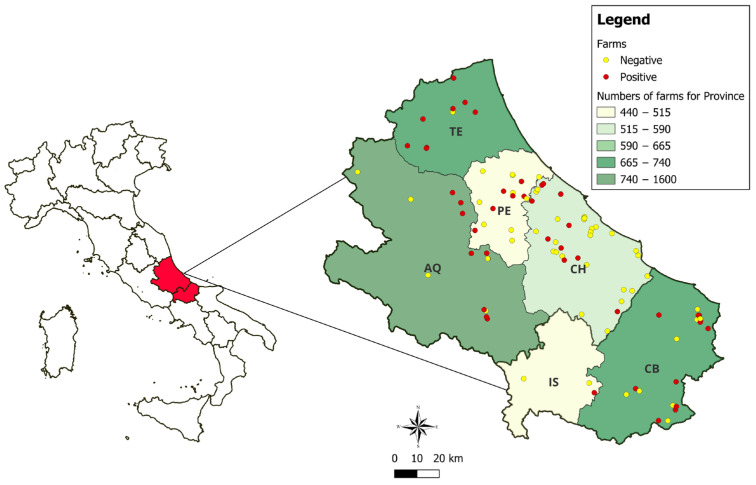
Geographical distribution of the farms under investigation, respectively, for the provinces of L’Aquila (AQ), Chieti (CH), Pescara (PE), and Teramo (TE), and Molise, in the provinces of Campobasso (CB) and Isernia (IS).

Of the samples collected from the 99 farms, 478 nasal swabs were analysed for the presence of SEZ. Of these farms, 56.6% had between 1 and 5 animals. Figure 1 shows the geographical distribution of the farms tested, highlighting those with positive results.

A total of 144 horses tested positive for SEZ by PCR, resulting in an estimated prevalence of 30.1% (95% CI: 26.2–34.4%) at the animal level. Of the 478 nasal swabs tested by culture, 56 (11.7%) were positive. All of these were also positive by PCR. At the herd level, 45 farms were classified as positive because at least one animal on the farm tested positive by PCR (*p* = 45.5%; 95% CI: 36.0–55.3%). The distribution of farms and the number of tested and positive animals by province is shown in Table 1. For four animals, information on the province of the sampled farm could not be traced. As a result, the number of tested animals (*n* = 474) and the number of positive animals (*n* = 143) in Table 1 do not correspond to the total number of sampled animals (*n* = 478) and the total number of positive animals (*n* = 144).

The logistic model was not significant, and none of the covariates considered reached significance (*p*-value > 0.05), as shown in Table 2. The results of the chi-square test were not statistically significant (*p*-value < 0.05) for all variables examined except for the species. Statistical analysis revealed a significant association between species and the presence of SEZ, with asinine being significantly more positive than equine (chi-square = 4.70 *p*-value = 0.030). The median age of positive animals was 6.5 years, significantly lower (*p*-value = 0.000, two tailed Mann–Whitney Test) than that of negative animals (median age: 10 years).

### 3.2. Genomic Characterisation and Cluster Analysis

**Figure 2:**
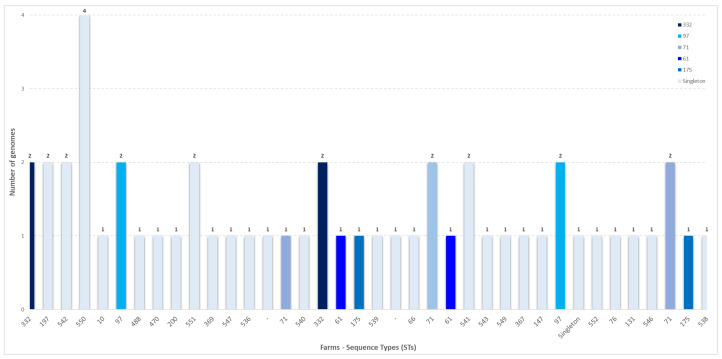
Sequence types identified across sampled farms, with each farm represented by a unique letter. Isolates identified as singletons are highlighted in light blue.

A total of 56 strains were isolated from nasal swabs. Of these, 51 underwent whole-genome sequencing (WGS), while 5 could not be sequenced due to poor DNA extraction quality. All the 51 genomes were confirmed to be SEZ. MLST analysis showed that the SEZ genomes could be classified into 31 sequence types (STs) (Table 3). Among the calculated STs, 14 new STs were identified: ST536, ST538, ST539, ST540, ST541, ST542, ST543, ST544, ST546, ST547, ST549, ST550, ST551, and ST552. MLST profiles could not be calculated for two isolates (2024.TE.15857.1.2 and 2024.TE.15855.1.2) due to the genome fragmentation. The 51 sequenced genomes came from 23 different farms, including 7 from Molise and 16 from Abruzzo. Figure 2 shows the farms and the sequence types identified in each one. In almost half of the farms (*n* = 11), more than one sequence type was detected.

The results of the virulence gene analyses are shown in Table 3. The *fbp54* gene, which codes for fibronectin-binding proteins, was present in 41 isolates. Meanwhile, *mf2* and *mf3* were identified in 19 and 29 strains, respectively. Interestingly, one isolate (2024.TE.6603.1.55) carried the *spel* gene, which encodes the precursor of streptococcal exotoxin L precursor, and the *spek* gene, which is involved in the production of streptococcal toxin associated with phages. The *hasC* gene, whose product is a cell wall surface anchor family protein, was found in 16 isolates.

**Figure 3:**
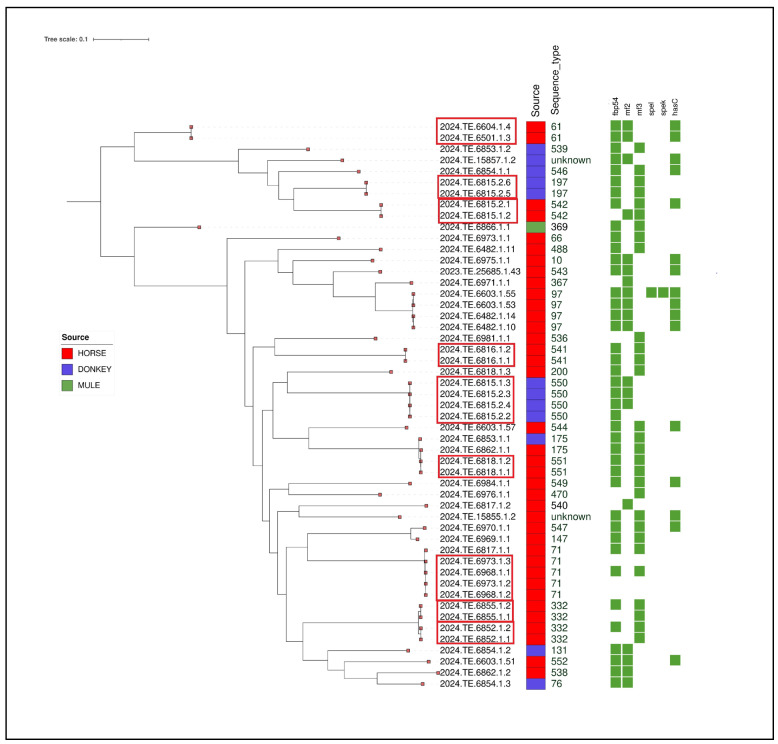
Maximum Likelihood (ML) midpoint-rooted tree obtained from CFSAN pipeline of the 51 SEZ isolates. The first layer represents the source of isolation as shown in the legend. The second layer represents MLST analysis results. Virulence genes are shown in the heatmap with a green colour. The clusters identified with SNP analysis were highlighted with a red box. The visualisation of genes profiles and genes presence/absence was visualised using the Interactive Tree of Life (iTOL) online tool (https://itol.embl.de/, accessed on 15 September 2024).

Clustering analysis highlighted the presence of different clusters according to MLST analysis (Figure 3). Specifically, a strain isolated from a donkey (2024.TE.6815.2.5) and belonging to ST197 clustered with another strain isolated from another donkey from the same farm. Two further clusters were identified on the same farm. Specifically, four strains isolated from donkeys belonged to cluster ST550 and two strains belonging to novel ST542 were isolated from two horses.

Another cluster was detected between two strains (ST541) isolated from two different horses belonging to the same breeder.

Furthermore, an SNP analysis revealed another cluster involving four SEZ strains (ST71) isolated from four horses sampled from two different farms in the provinces of Chieti and L’Aquila; meanwhile, another strain (2024.TE.6817.1.1) belonging to ST71, isolated from another farm in the province of L’Aquila, was distant from the rest of the cluster. No epidemiological link was found between the three farms.

A similar situation was observed for the ST332 strains. Specifically, one cluster was identified for two strains isolated from two horses belonging to the same farm in the province of Isernia, while the isolated strain (2024.TE.6852.1.1) was correlated with another strain (2024.TE.6852.1.2), both originating from two horses belonging to the same farm in the province of Campobasso. Furthermore, a cluster (ST541) was identified between two horses sampled from the same farm in the province of Teramo.

Another cluster was identified between two strains belonging to ST551 and isolated from two different horses.

Finally, two strains (ST61) isolated from two different horses on the same farm in the province of Campobasso were correlated. Given the relevance of this clone following the outbreak in the “Vestina” area in the period November 2021–May 2022 [8], a comparative genomics analysis was performed on these two strains with those belonging to the aforementioned outbreak. The results showed that the strains isolated in this study are not correlated. Animal movement records also that there were no connections between the farm in Campobasso and the farm where the outbreak occurred.

The remaining isolates were singletons.

## 4. Discussion

*Streptococcus equi* subsp. *zooepidemicus* (SEZ) is a commensal bacterium found in the pharyngeal mucosa of various animal species. Studies of SEZ in equids are mostly limited to post-symptomatic observations or seroprevalence data, where the detection of antibodies indicates prior exposure but does not confirm carrier status. However, the literature does not allow for population-wide inferences, as it often relies on opportunistic sampling. The only probabilistic study, conducted by Libardoni et al. in Brazil [17] and focusing on estimating the prevalence of *Streptococcus equi* subsp. *equi*, reported a prevalence of 2.37% at the animal level and 5.86% at the herd level.

This study not only assesses the prevalence of SEZ in equine populations in the regions of Abruzzo and Molise (Italy), but also investigates the risk factors associated with the presence of SEZ and the genetic characteristics of the circulating SEZ strains. Traditional strain typing methods do not capture the genomic diversity of SEZ and only *Streptococcus equi* subsp. *zooepidemicus* from other streptococci. Following a local human outbreak associated with SEZ from raw milk cheeses [8] and the emergence of a highly virulent strain that caused significant mortality in a donkey herd [9], it became essential to assess whether these strains differed genetically from typical circulating strains.

Our results show a prevalence of SEZ of 30.1% at the animal level and 45.5% at the herd level, which is significantly higher than the prevalence of *Streptococcus equi* subsp. *equi* reported by Libardoni et al. [17], despite our smaller sample size. The higher percentage of animals sampled in this study (456/23,588 versus 1010/522,578 in the Brazilian study) may account for this difference, together with the fact that SEZ is considered a commensal bacterium, unlike *Streptococcus equi* subsp. *equi*. Notably, our multivariable logistic regression analysis showed no significant associations between the risk factors studied and the presence of SEZ. Variables such as respiratory symptoms or the presence of dairy species on the farm did not correlate with SEZ prevalence, suggesting that pharyngeal SEZ colonisation does not necessarily lead to respiratory symptoms. While some variables, such as age and species, showed significant associations in univariable analyses (e.g., chi-square and Mann–Whitney tests), these associations did not persist in the multivariable model. There may be several reasons for this discrepancy. First, the inclusion of multiple predictors, some of which may be correlated or weakly associated with the outcome, may have introduced multicollinearity and diluted the apparent effects of individual variables. Second, the true effect sizes of certain variables may be modest and require a larger sample size to be detected with adequate statistical power in a multivariable context. Third, unmeasured confounders or effect modifiers—such as variations in animal health status, management practices, or environmental exposures—may have masked potential associations. In addition, intermittent bacterial shedding and inconsistencies in sampling techniques between independent professionals may have affected detection rates.

Interestingly, donkeys had a significantly higher prevalence of SEZ than horses. This may reflect the role of donkeys as asymptomatic carriers, as they are generally susceptible to respiratory pathogens, although they often present with milder symptoms than horses. This species-specific susceptibility highlights a potential area for further research, particularly to identify mechanisms that could inform more tailored management practices for donkeys to limit SEZ transmission.

Another important observation is the significantly younger age of SEZ-positive animals compared to negative animals, suggesting age-related susceptibility factors. This association invites further investigation into the genetic, environmental, and nutritional influences on younger animals, which may help to improve management strategies across different age groups.

A genomic analysis revealed a high degree of variability among circulating strains. Fourteen new STs were identified, and these results highlight the importance of using whole-genome sequencing (WGS) to characterise SEZ strains, contributing to the expanding knowledge of this still poorly understood pathogen, as shown by Nocera et al., 2023 [18]. The remaining seventeen had been previously reported. In particular, ST200 and ST10 had been detected in cervico-uterine swabs from mares with endometritis [18], as well as in both healthy dogs and those with respiratory symptoms [19,20]. In contrast, ST147 was previously isolated from a horse breeding facility in China and from a mare in Argentina in 2017 [21,22]. ST71 has been isolated from the respiratory tract of horses in the UK and USA over an extended period [23]. ST369 was detected in cervico-uterine swabs from mares with endometritis in Italy [18].

Finally, ST61 was isolated from the clitoris of a mare in 2013 [24]. Although this clone has been identified as the source of local human outbreaks [8], the strains isolated in this study and belonging to the same sequence type did not show any correlation with those previously mentioned.

The fact that the identified genotypes have also been reported in different countries and host species suggests that there is no specific adaptation to a particular host.

Emerging clones such as ST194 and ST132 were not isolated in this study. These two clones have been associated with several epidemic outbreaks in different regions of the world. ST194 was first documented in a swine mortality outbreak in China in the 1970s [25], where the ATCC 35246 strain was responsible for a large-scale infection that resulted in the death of over 300,000 pigs. Subsequently, this ST has been implicated in significant outbreaks in North America, notably in Ohio and Tennessee in 2019 [26], where high mortality rates were observed in pig populations. Although ST132 has been less extensively studied, it has also been identified in outbreaks predominantly affecting livestock, with notable public health and economic impacts.

Genomic characterisation revealed a significant similarity to group A streptococci (GAS), particularly those associated with GAS infections. Most SEZ strains carried the *fpb54* gene, which encodes a streptococcal fibronectin-binding protein—a critical factor in GAS infection, facilitating attachment to human buccal epithelial cells [27,28]. In addition, 19 of the 51 SEZ strains carried the *mf2* gene, a virulence factor associated with *Streptococcus pyogenes* prophages that is rarely detected in SEZ [9]. Another GAS-associated virulence factor, mf3, encoding mitogen factor 3 (MF3) with DNase activity, has also been identified [29].

In one isolate, both the spel and spek genes associated with streptococcal exotoxin production were detected. The *spel* gene, first detected in SEZ in 2005, shares over 98% identity with the spel gene of *S. pyogenes*, suggesting possible horizontal gene transfer. The superantigenic toxin encoded by the *spel* gene is a common virulence factor in *S. equi* subsp. *equi*, but is less common in SEZ [30]. The *spek* gene, which is highly similar to the *seeL* gene, plays a role in immune evasion by suppressing phagocyte recruitment [31]. Consequently, isolate 2024.TE.6603.1.55 has considerable virulence potential.

Finally, this study confirmed the presence of the *hasC* gene from the has operon, encoding UDP-glucose dehydrogenase, which is required for the synthesis of the SEZ hyaluronic acid capsule [32]. This capsule, which mimics human connective tissue, reduces the host immune response and represents another virulence mechanism [33].

## 5. Conclusions

Overall, this study provides valuable insights into distribution of SEZ in the target regions, showing considerable strain variability and a higher prevalence in donkeys. The lack of association with cohabitation with dairy species contradicts previous assumptions about risk factors. These findings highlight the need for further research to determine species-specific susceptibility mechanisms and to identify factors that activate SEZ virulence, which could have implications for both animal and public health.

## Figures and Tables

**Table 1:** Distribution of farms and animals sampled and number of testing positive by PCR for Abruzzo, in the provinces of L’Aquila (AQ), Chieti (CH), Pescara (PE), and Teramo (TE), and Molise, in the provinces of Campobasso (CB) and Isernia (IS).

Province	Total Herds	Total Animals	Positive Herds (Number of Positive Herds/ Number of Herds Tested)	Positive Animals (Number of Positive Animals/ Number of Animals Tested) *
AQ	1866 (41%)	12,142 (58%)	64% (9/14)	36% (52/145)
CH	624 (14%)	1950 (9%)	26% (10/38)	17% (13/78)
PE	552 (12%)	2043 (10%)	40% (6/15)	28% (14/50)
TE	802 (18%)	2796 (13%)	89% (8/9)	29% (45/155)
CB	725 (16%)	2065 (10%)	55% (11/20)	40% (16/40)
IS	481 (11%)	2592 (12%)	33% (1/3)	50% (3/6)
Total	5050 (100%)	23,588 (100%)	46% (45/99)	30% (143/474)

* The number of animals tested and the number of animals that tested positive in Table 1 differ from those detailed in the text of the paper, considering that for four animals, the information regarding the province of the farm sampled is not present.

**Table 2:** Results of the covariate significance testing (null deviance: 499.11 on 402 degrees of freedom; residual deviance: 488.47 on 395 degrees of freedom; AIC: 504.47).

Coefficients	Estimate	Std. Error	z Value	Pr (>|z|)
(Intercept)	−0.43583	0.35862	−1.215	0.224
Sex M	−0.11497	0.22638	−0.508	0.612
Species Equine	−0.43152	0.26911	−1.603	0.109
Breed YES	0.17963	0.32104	0.560	0.576
Symptoms YES	−0.41085	0.53616	−0.766	0.444
Dairy species YES	0.32565	0.22357	1.457	0.145
Intended for food production YES	−0.08249	0.24038	−0.343	0.731
Age_category YOUNG	−0.47630	0.28268	−1.685	0.092

**Table 3:** Metadata and genomic characteristics of 51 SEZ strains.

ID	Source	Province	ST	Fbp54	mf2	mf3	spel	spek	hasC	Biosample
2024.TE.6815.2.1	Horse	Teramo	542	Presence	Absence	Presence	Absence	Absence	Presence	SAMN48192347
2024.TE.6815.1.2	Horse	Teramo	542	Absence	Presence	Presence	Absence	Absence	Absence	SAMN48192348
2024.TE.6815.2.2	Donkey	Teramo	550	Presence	Absence	Absence	Absence	Absence	Absence	SAMN48192349
2024.TE.6815.1.3	Donkey	Teramo	550	Presence	Presence	Absence	Absence	Absence	Absence	SAMN48192350
2024.TE.6815.2.3	Donkey	Teramo	550	Presence	Presence	Absence	Absence	Absence	Absence	SAMN48192351
2024.TE.6815.2.4	Donkey	Teramo	550	Presence	Presence	Absence	Absence	Absence	Absence	SAMN48192352
2024.TE.6815.2.5	Donkey	Teramo	197	Presence	Absence	Presence	Absence	Absence	Absence	SAMN48192353
2024.TE.6815.2.6	Donkey	Teramo	197	Presence	Absence	Presence	Absence	Absence	Absence	SAMN48192354
2024.TE.6816.1.1	Horse	Teramo	541	Presence	Absence	Presence	Absence	Absence	Absence	SAMN48192355
2024.TE.6816.1.2	Horse	Teramo	541	Presence	Absence	Presence	Absence	Absence	Absence	SAMN48192356
2023.TE.25685.1.43	Horse	Teramo	543	Presence	Presence	Absence	Absence	Absence	Presence	SAMN48192357
2024.TE.6817.1.1	Horse	L’Aquila	71	Presence	Absence	Presence	Absence	Absence	Absence	SAMN48192358
2024.TE.6817.1.2	Horse	L’Aquila	540	Absence	Presence	Absence	Absence	Absence	Absence	SAMN48192359
2024.TE.6818.1.1	Horse	Teramo	551	Presence	Absence	Presence	Absence	Absence	Absence	SAMN48192360
2024.TE.6818.1.2	Horse	Teramo	551	Presence	Absence	Presence	Absence	Absence	Absence	SAMN48192361
2024.TE.6818.1.3	Horse	Teramo	200	Presence	Absence	Presence	Absence	Absence	Absence	SAMN48192362
2024.TE.6852.1.1	Horse	Campobasso	332	Absence	Absence	Presence	Absence	Absence	Absence	SAMN48192363
2024.TE.6852.1.2	Horse	Campobasso	332	Presence	Absence	Presence	Absence	Absence	Absence	SAMN48192364
2024.TE.6853.1.1	Donkey	Pescara	175	Presence	Absence	Presence	Absence	Absence	Absence	SAMN48192365
2024.TE.6853.1.2	Donkey	Pescara	539	Presence	Absence	Presence	Absence	Absence	Absence	SAMN48192366
2024.TE.6854.1.1	Donkey	Teramo	546	Presence	Absence	Presence	Absence	Absence	Presence	SAMN48192367
2024.TE.6854.1.2	Donkey	Teramo	131	Presence	Presence	Absence	Absence	Absence	Absence	SAMN48192368
2024.TE.6854.1.3	Donkey	Teramo	76	Presence	Presence	Absence	Absence	Absence	Absence	SAMN48192369
2024.TE.6855.1.1	Horse	Isernia	332	Absence	Absence	Presence	Absence	Absence	Absence	SAMN48192370
2024.TE.6855.1.2	Horse	Isernia	332	Presence	Absence	Presence	Absence	Absence	Absence	SAMN48192371
2024.TE.6970.1.1	Horse	Pescara	547	Presence	Absence	Presence	Absence	Absence	Presence	SAMN48192372
2024.TE.6862.1.1	Horse	Teramo	175	Presence	Absence	Presence	Absence	Absence	Absence	SAMN48192373
2024.TE.6862.1.2	Horse	Teramo	538	Presence	Presence	Absence	Absence	Absence	Absence	SAMN48192374
2024.TE.6866.1.1	Mule	Pescara	369	Presence	Absence	Presence	Absence	Absence	Absence	SAMN48192375
2024.TE.6968.1.1	Horse	Chieti	71	Presence	Absence	Presence	Absence	Absence	Absence	SAMN48192376
2024.TE.6968.1.2	Horse	Chieti	71	Absence	Absence	Absence	Absence	Absence	Absence	SAMN48192377
2024.TE.6969.1.1	Horse	Chieti	147	Presence	Absence	Presence	Absence	Absence	Absence	SAMN48192378
2024.TE.6971.1.1	Horse	Teramo	367	Absence	Presence	Absence	Absence	Absence	Absence	SAMN48192379
2024.TE.6973.1.1	Horse	L’Aquila	66	Presence	Absence	Presence	Absence	Absence	Absence	SAMN48192380
2024.TE.6973.1.2	Horse	L’Aquila	71	Absence	Absence	Absence	Absence	Absence	Absence	SAMN48192381
2024.TE.6973.1.3	Horse	L’Aquila	71	Absence	Absence	Absence	Absence	Absence	Absence	SAMN48192382
2024.TE.6975.1.1	Horse	Chieti	10	Presence	Presence	Absence	Absence	Absence	Presence	SAMN48192383
2024.TE.6976.1.1	Horse	Campobasso	470	Absence	Absence	Presence	Absence	Absence	Absence	SAMN48192384
2024.TE.6981.1.1	Horse	Campobasso	536	Absence	Absence	Presence	Absence	Absence	Absence	SAMN48192385
2024.TE.6984.1.1	Horse	Teramo	549	Presence	Absence	Presence	Absence	Absence	Presence	SAMN48192386
2024.TE.15857.1.2	Donkey	L’Aquila	unknown	Presence	Presence	Absence	Absence	Absence	Presence	SAMN48192387
2024.TE.6603.1.51	Horse	L’Aquila	552	Presence	Presence	Absence	Absence	Absence	Presence	SAMN48192388
2024.TE.6482.1.11	Horse	Campobasso	488	Presence	Absence	Presence	Absence	Absence	Absence	SAMN48192389
2024.TE.6482.1.10	Horse	Campobasso	97	Presence	Presence	Absence	Absence	Absence	Presence	SAMN48192390
2024.TE.6482.1.14	Horse	Campobasso	97	Presence	Presence	Absence	Absence	Absence	Presence	SAMN48192391
2024.TE.6603.1.57	Horse	L’Aquila	544	Presence	Absence	Presence	Absence	Absence	Presence	SAMN48192392
2024.TE.6603.1.53	Horse	L’Aquila	97	Presence	Presence	Absence	Absence	Absence	Presence	SAMN48192393
2024.TE.15855.1.2	Horse	L’Aquila	unknown	Presence	Absence	Presence	Absence	Absence	Presence	SAMN48192394
2024.TE.6603.1.55	Horse	L’Aquila	553	Presence	Presence	Absence	Presence	Presence	Presence	SAMN48192395
2024.TE.6501.1.3	Horse	Campobasso	61	Presence	Presence	Absence	Absence	Absence	Presence	SAMN48192396

## Data Availability

All sequences are publicly available in the National Center for Biotechnology Information (NCBI) database. The accession numbers are detailed in Table 3.

## Abbreviations

The following abbreviations are used in this manuscript:

SEZ	Streptococcus equi subsp. zooepidemicus
IZSAM	Istituto Zooprofilattico Sperimentale dell’Abruzzo e Molise
WGS	Whole Genome Sequencing
